# Machine Learning‐Driven Cooling Window Design Beyond Hyperbolic Metamaterials

**DOI:** 10.1002/nap2.70028

**Published:** 2026-02-21

**Authors:** Seok‐Beom Seo, Ye‐Rin Choi, Jong‐Goog Lee, Gumin Kang, Hyungduk Ko, Run Hu, Sun‐Kyung Kim

**Affiliations:** ^1^ Department of Applied Physics Kyung Hee University Yongin South Korea; ^2^ Nanophotonics Research Center Korea Institute of Science and Technology Seoul South Korea; ^3^ KHU‐KIST Department of Converging Science and Technology Kyung Hee University Seoul South Korea; ^4^ School of Energy and Power Engineering Huazhong University of Science and Technology Wuhan China

**Keywords:** energy saving, hyperbolic metamaterial, machine learning outperformance, metal/dielectric multilayer, optical coating, passive cooling

## Abstract

Analytical multilayers designed under quarter‐wave conditions, such as antireflective coatings and distributed Bragg reflectors, generally perform effectively within narrow spectral bands but often face challenges in meeting multispectral demands. In contrast, machine learning (ML)‐driven inverse design enables exploration of vast parameter spaces to realize tailored spectral responses across multiple bands. However, whether ML‐optimized multilayers can outperform analytical designs under identical material and thickness constraints often remains an open question. Here, we experimentally validate the superiority of ML‐driven design through a metal/dielectric multilayer cooling‐window coating that simultaneously requires high average visible transmittance (AVT) and high average near‐infrared reflectance (ANR). By integrating a factorization machine with simulated annealing, we discovered optimized aperiodic ZnS/Ag multilayers and benchmarked them against periodic hyperbolic metamaterial (HMM) counterparts. Under a 156 nm thickness constraint (equivalent to two ZnS/Ag pairs in a HMM), the ML design achieved 0.57 AVT and 0.98 ANR, surpassing the HMM reference (0.49 AVT, 0.83 ANR). With an extended thickness of 300 nm, the ML‐optimized coating further improved to 0.79 AVT by suppressing Fabry–Perot resonances while maintaining high ANR (0.97). Furthermore, the ML‐driven multilayers exhibited tunable transmitted colors spanning the full visible gamut, whereas the HMM counterparts were restricted to specific hues. Both ML and HMM designs were fabricated on glass, and measured spectra confirmed the superior optical and thermal performance of the ML approach. These findings establish ML‐driven inverse design as a powerful route to ultrathin, manufacturable, and color‐tunable cooling‐window coatings that can contribute to urban energy savings.

## Graphical abstract

Machine learning‐driven inverse design enables ultrathin metal/dielectric cooling‐window coatings that outperform analytical hyperbolic metamaterials under identical material and thickness constraints. Optimized aperiodic multilayers simultaneously enhance visible transparency, near‐infrared rejection, and color tunability, demonstrating a practical pathway toward high‐performance, manufacturable, and energy‐efficient photonic coatings.

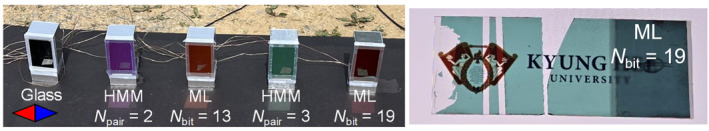

## 1 Introduction

One‐dimensional multilayers represent the most versatile and widely used classes of optical coatings, with applications ranging from extreme‐ultraviolet (EUV) lithography, where distributed Bragg reflectors (DBRs) are indispensable, to mid‐infrared radiative coolers [1]. Such multilayers enable diverse functionalities, including antireflection and antiglare coatings [2, 3], reflectors [4, 5, 6, 7], absorbers [8, 9, 10, 11], bandpass and bandstop filters [12], anticounterfeiting elements [13, 14], and optical camouflage [15]. They are typically realized by stacking two or more materials at subwavelength scales, most often designed according to the quarter‐wave interference principle. For example, a periodic quarter‐wave stack provides nearly perfect reflection within a target band, with the bandwidth determined by the refractive index contrast of its constituent materials.

Although such physics‐rooted designs are effective for single‐band objectives, they struggle to satisfy growing demands for multifunctional, multispectral performance—simultaneously regulating transmission, reflection, or absorption across different bands. Tandem multilayer stacks tailored for separate bands can partially address these requirements but at the expense of excessive thickness, greater fabrication complexity, increased material usage, and unpredictable interference. Recent advances in machine learning (ML) provides a promising alternative by enabling inverse design of nonintuitive, aperiodic multilayers optimized across multiple objectives simultaneously. Notable demonstrations include Bayesian optimization of aperiodic emitters with ultranarrow thermal emissivity [7] and exceptional epsilon‐based microcavities with directional thermal emission [9]. These studies illustrate that data‐driven optimization can effectively navigate complex design spaces that are difficult to address with conventional analytical methods. Inverse design of dual‐band camouflage using a materials‐informatics framework [15], and neural‐network‐driven inverse design of variable‐index multilayers incorporating fabrication‐error tolerance [12].

Passive cooling windows impose stringent multispectral requirements: high average visible transmittance (AVT, 380–780 nm) to admit daylight and high average near‐infrared reflectance (ANR, 780–2500 nm) to suppress indoor heat gain. As modern buildings and vehicles increasingly adopt large glass facades, such coatings can significantly reduce cooling loads and enhance urban energy resilience [16]. Current cooling window strategies fall into two categories: all‐dielectric [17, 18, 19] and metal/dielectric multilayers [20, 21, 22, 23, 24]. In the realm of all‐dielectric structures, D. O. Ruiz et al. recently demonstrated that AI‐enhanced optimization can yield metamaterial Bragg multilayers superior to analytical designs [7], validating the efficacy of data‐driven approaches. All‐dielectric multilayers typically achieve high AVT but are limited in ANR due to the finite refractive index contrast of dielectric materials. For example, Hu's group reported a ZnS/MgF_2_ five‐layer stack (550 nm total thickness), yielding 0.93 AVT and 0.48 ANR [17]. Luo's group expanded the space to 20 layers using four dielectric materials (SiO_2_/Al_2_O_3_/Si_3_N_4_/TiO_2_) and a total thickness of 1.2 μm, achieving 0.92 AVT and 0.62 ANR with the aid of a quantum annealer [18]. These studies underscore that increased structural complexity through greater total thickness and material diversity can improve ANR, yet fundamental trade‐offs remain.

Metal/dielectric periodic multilayers, or hyperbolic metamaterials (HMMs), provide an alternative route. Within the effective medium approximation, HMMs undergo an elliptic‐to‐hyperbolic dispersion transition at an epsilon‐near‐zero (ENZ) wavelength, marking the cutoff wavelength (e.g., ∼780 nm for cooling window applications) between transmission and reflection [20, 21]. For example, Yu's group reported a SiO_2_/Ag HMM with a 441 nm total thickness, achieving ∼0.6 AVT and 0.89 ANR [22]. However, HMMs are constrained by strict periodicity, and their spectral performance is not always optimal under realistic thickness and material limits. In contrast, ML‐driven inverse design can explore a much broader parameter space to identify aperiodic metal/dielectric configurations tailored for simultaneous AVT–ANR optimization. For example, Rho's group employed alternating TiO_2_/SiO_2_ with a single Ag layer, achieving ≈0.60 AVT and 0.80 ANR at 271 nm thickness [23]. Cui's group combined Al_2_O_3_, HfO_2_, SiO_2_, and an Ag–Ge alloy to reach 0.75 AVT and 0.93 ANR at 637 nm thickness [24]. Despite these advances, most ML‐based cooling windows rely on diverse material sets or relaxed thickness budgets relative to DBR or HMM baselines, leaving open a key question: Can ML‐driven multilayers still outperform analytical designs under identical material choices and thickness constraints?

In this work, we address this question by directly benchmarking ML‐driven multilayers against HMMs design for cooling windows under identical conditions. Based on our prior demonstration of ML‐optimized all‐dielectric ZnS/MgF_2_ multilayers that outperformed DBRs under strict thickness constraints, we extended the approach to a metal/dielectric platform. Specifically, we focused on ZnS/Ag multilayers and compare ML‐optimized designs with HMMs at nearly equal total thicknesses (150–375 nm, corresponding to 2–5 pairs in HMMs). Using a factorization machine (FM) integrated with simulated annealing (SA), we identified optimized aperiodic multilayers with varying discretization levels (13–31 bits, each representing 12 nm) that maximize visible transparency while maintaining strong near‐infrared reflection. Furthermore, we examined the color‐tuning capability of ML‐driven multilayers in comparison with their HMM counterparts. Using experimentally derived optical constants, we fabricated representative ML designs (13‐bit and 19‐bit multilayers) and their corresponding HMMs, and experimental spectra confirmed the superior optical and thermal performance of the ML‐driven designs. These results establish ML‐driven inverse design as a practical route to ultrathin, economically viable, and aesthetic cooling‐window coatings that advance both nanophotonic design methodology and sustainable energy technologies.

## 2 Results and Discussion

### 2.1 Benchmark Problem Definition

**FIGURE 1:**
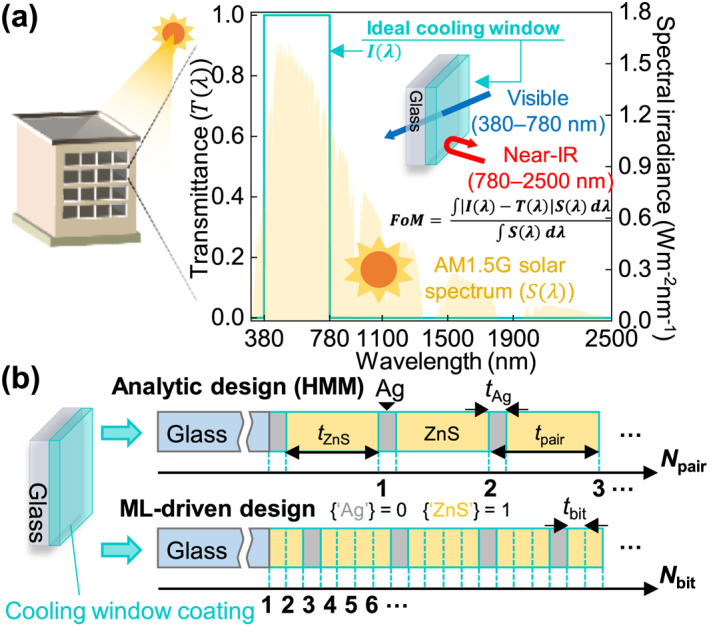
Benchmark problem definition. (a) Target spectrum for passive cooling windows. Within the AM 1.5G solar spectrum (300–2500 nm), the coating is designed to achieve the ideal transmittance–reflectance profile indicated by the cyan curve, exhibiting high transmittance in the visible and high reflectance in the near‐infrared region. (b) Schematic of benchmarked multilayer configurations. In the analytical HMM design, ZnS and Ag layers with prescribed thicknesses are periodically stacked. In the ML‐driven design, each layer is encoded as a binary vector with a unit thickness (*t*_bit_), where 0 and 1 represent Ag and ZnS, respectively, enabling aperiodic configurations under the same total thickness constraint.

To demonstrate the superiority of ML in multilayer design, we employed the cooling window problem as a benchmark. This benchmark problem inherently requires multispectral optimization, as the entire AM1.5G solar spectrum (300–2500 nm) must be considered. To achieve high AVT and high ANR across this range, we defined an ideal cooling‐window spectrum, *I*(λ), as shown in Figure 1a. The deviation between this ideal response and the transmittance spectrum of a given multilayer design was quantified by a figure of merit (FoM)—a central metric in our ML framework that measures how closely a design approaches the target spectrum. A lower FoM thus indicates a transmittance spectrum that better matches the ideal response.

Both the analytical HMM and the ML‐driven design were optimized under identical materials and closely matched total thickness constraints, as schematized in Figure 1b. In the ML‐driven approach, each multilayer configuration was encoded as a binary vector consisting of 0 and 1s, where one bit corresponds to a unit thickness (*t*_bit_), and the total number of bits (*N*_bit_) was chosen to match the total thickness of the reference HMM. The ML algorithm determined whether each bit was 0 or 1, corresponding to Ag (0) and ZnS (1), respectively, thereby defining an aperiodic multilayer. In contrast, the HMM configuration consisted of periodically repeating Ag and ZnS layers with predetermined thicknesses (*t*_Ag_ and *t*_ZnS_) derived from analytical design principles discussed in the following section. The total thickness was thus an integer multiple of the pair thickness (*t*_pair_), determined by the number of ZnS/Ag pairs (*N*_pair_).

### 2.2 Design Principle of Baseline HMMs

**FIGURE 2:**
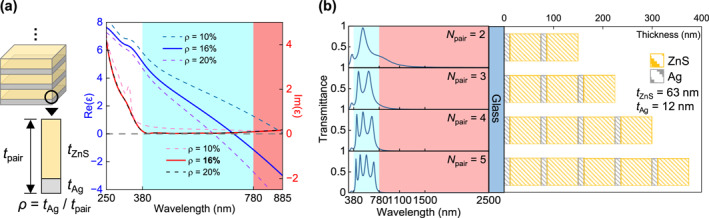
Design principle of baseline HMMs. (a) Effective permittivity of ZnS/Ag multilayers as a function of Ag filling fraction (*ρ*). At *ρ* = 16%, the real part of the effective permittivity crosses zero near 780 nm, marking the dielectric‐to‐metal transition. (b) Calculated transmittance spectra (left) and corresponding layer configurations (right) of HMMs with varying numbers of ZnS/Ag pairs (*N*_pair_).

The baseline HMM was designed based on effective medium theory. For alternating metal–dielectric stacks, a dielectric‐to‐metal transition occurs at the epsilon‐near‐zero (ENZ) wavelength, where the real part of the effective permittivity crosses zero. Placing this ENZ point near 780 nm enables a HMM suitable for cooling window operation. The corresponding Ag filling fraction (*ρ*) was determined to be 16% (Figure 2a). The permittivity values of the constituent materials were obtained from ellipsometry measurements on ultrathin Ag and ZnS films (Supporting Information S1: Figure S1). The remaining choice design variable was *t*_Ag_. Although thinner Ag layers theoretically improve optical performance, we fixed *t*_Ag_ = 12 nm to avoid percolation issues typical of vacuum‐deposited Ag films. This choice yielded *t*_ZnS_ = 63 nm and a total period thickness of *t*_pair_ 75 nm. The resulting HMM effectively suppressed near‐infrared transmission while maintaining moderate visible transparency; an ANR approached unity at *N*_pair_ = 5 (Figure 2b). Notably, in the visible region, the number of transmission peaks equaled *N*_pair_, indicating that the HMM behaves as a multimodal Fabry–Perot cavity.

### 2.3 ML‐Driven Design Strategy and Outperformance

**FIGURE 3:**
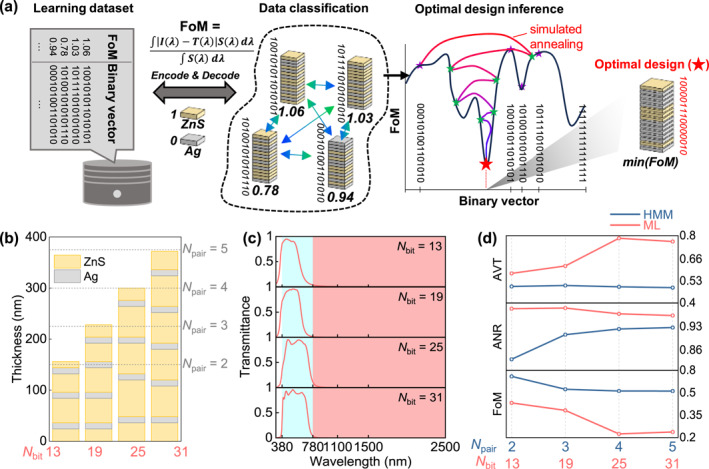
ML design workflow and benchmarking against HMMs. (a) Schematics of inverse design processes. Candidate multilayers are encoded as binary vectors (0 = Ag, 1 = ZnS) and evaluated by FoM. The red star indicates the optimal binary vector with minimum FoM obtained by the FM combined with SA. (b) Configurations of the four ML‐driven designs (*N*_bit_ = 13, 19, 25, and 31) aligned with their HMM counterparts (*N*_pair_ = 2, 3, 4, and 5). (c) Calculated transmittance spectra of the ML‐driven multilayers. (d) Quantitative comparison of AVT, ANR, and FoM between ML and HMM designs under identical thickness constraints.

To overcome the inherent limitations of analytical multilayers, we employed a ML‐driven inverse design framework based on a FM integrated with simulated annealing (SA) (Figure 3a). This hybrid approach offers two distinct advantages over conventional neural‐network or gradient‐based optimizers. First, the FM efficiently captures nonlinear correlations among discrete design parameters even with a limited dataset, which is ideal for combinatorial multilayer design spaces. Second, the SA introduces stochastic exploration that avoids local minima, enabling the identification of nearly optimal aperiodic configurations. Together, the FM–SA pipeline rapidly converges toward multilayer architectures that minimize the FoM, which quantifies the deviation between the designed and target spectra. Each active learning run starts from 25 random designs and adds one evaluated design per cycle (2000 cycles; 2025 evaluated designs total).

Although the FM–SA pipeline may appear more complex, its direct compatibility with the quadratic unconstrained binary optimization (QUBO) formulation allows users to inspect the learned QUBO coefficients and infer how specific bits (and their pairwise couplings) influence the overall FoM. Moreover, this added interpretability does not come at the expense of optimization quality; the NSGA II benchmark reaches solution qualities comparable to those obtained by FM–SA, indicating that FM–SA remains competitive with standard heuristic optimizers (Supporting Information S1: Figure S2) [25].

Using this framework, we derived four optimized ZnS/Ag multilayers with *N*_bit_ = 13, 19, 25, and 31, corresponding to the HMMs with *N*_pair_ = 2, 3, 4, and 5 (Figure 3b). Each bit represented a 12 nm unit thickness (*t*_bit_ = 12 nm) and the binary sequence (0 = Ag, 1 = ZnS) defined the stacking order. Notably, the ML designs with *N*_bit_ = 13 and 19 contained one additional Ag layer compared with their HMM counterparts, revealing that the ML optimization fundamentally deviated from the periodic HMMs governed by effective medium theory.

The resulting spectral characteristics confirmed the structural distinction (Figure 3c). The ML‐driven multilayers eliminated the multiple Fabry–Perot peaks that were evident in the visible region of the HMM spectra, yielding flattened high‐transmission profiles across 380–780 nm. Simultaneously, the transmission was sharply suppressed beyond the cutoff wavelength (780 nm), the design with *N*_bit_ = 19 exhibiting near‐unity ANR. These results demonstrate that the ML optimization effectively redistributes the internal optical phasors to suppress undesired resonances while maintaining the targeted near‐infrared reflection [3].

Quantitative benchmarking showed that under identical total thickness constraints, all ML‐driven designs achieved higher AVT and ANR than the corresponding HMMs (Figure 3d). Remarkably, for *N*_bit_ = 13 and 19, the ML designs exhibited higher AVT despite including an additional Ag layer, underscoring their trade‐off management between visible transparency and near‐infrared reflectance. The reduced FoM across all cases confirmed the supremacy of the ML approach in both optical and thermal performance.

### 2.4 Outdoor Experimental Validation of ML‐Driven Coating

**FIGURE 4:**
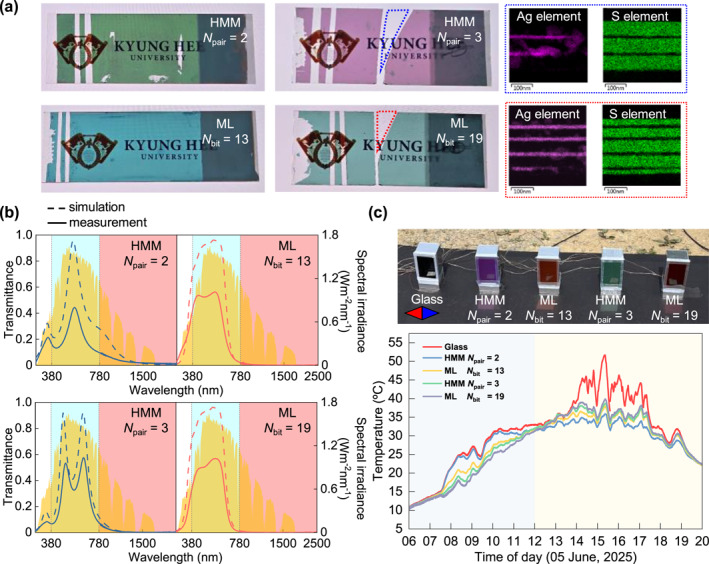
Experimental validation of ML‐driven coating outperformance. (a) Photographs of fabricated glass samples: HMMs (*N*_pair_ = 2 and 3) and ML‐driven coatings (*N*_bit_ = 13 and 19). Inset: cross‐sectional EDS elemental maps (Ag and S) of *N*_pair_ = 3‐HMM and *N*_bit_ = 19‐ML coatings, showing Ag_2_S formation. (b) Measured (solid) and simulated (dashed) transmittance spectra of the fabricated coatings. (c) Outdoor sunlight test. Experimental setup (top) and recorded interior temperatures (bottom) for uncoated and coated glass samples under sunlight.

To experimentally validate the predicted ML outperformance, we fabricated representative multilayers and evaluated their optical and thermal performance (Figure 4a). Two ML‐driven coatings (*N*_bit_ = 13 and 19) and their corresponding HMM counterparts (*N*_pair_ = 2 and 3) were deposited on glass substrates through thermal deposition under identical conditions. Optical spectra were recorded using UV–vis–NIR spectrophotometry (Figure 4b).

The measured transmittance and reflectance closely reproduced the simulated trends. Specifically, the *N*_bit_ = 13‐ and 19‐ML coatings exhibited AVT of 0.33 and 0.35 and ANR of 0.98 and 0.97, respectively. In contrast, the corresponding HMMs yielded AVT = 0.23 and 0.33 with ANR = 0.73 and 0.84. Detailed performance metrics are provided in Supporting Information S1: Figure S3. These measurements confirm that the ML‐driven multilayers achieved higher near‐infrared rejection with enhanced visible transparency relative to their periodic counterparts. A key indicator of ML design's success is the consistent suppression of Fabry–Perot resonance peaks in the visible region, which are prominent in the HMM counterparts.

A modest reduction in measured transmittance amplitude compared with simulation is attributed to interfacial degradation in the deposited films. Cross‐sectional transmission electron microscopy (TEM) and energy‐dispersive X‐ray spectroscopy (EDS) analyses revealed partial Ag migration and Ag_2_S formation at ZnS interfaces, causing additional absorption (Figure 4a, inset). Because this interfacial effect appeared in both ML and HMM samples, the comparative advantage of the ML‐driven design remains valid. The optical trends observed strongly suggest that using chemically stable dielectrics with comparable refractive indices (e.g., TiO_2_) would eliminate Ag migration and restore the simulated AVT levels without compromising the ML‐optimized spectral selectivity (Supporting Information S1: Figure S4).

To further assess real‐world cooling performance, all coated and uncoated glass samples were installed as vertical façades in miniature test houses, and interior temperatures were continuously monitored under outdoor sunlight (Figure 4c). To minimize experimental errors from environmental variables such as wind speed and solar incidence angle, all coated and uncoated samples were measured simultaneously in a side‐by‐side configuration. Starting from noon, when direct illumination reached the samples, all coated windows exhibited lower indoor temperatures than those of uncoated glass, consistent with their reduced solar transmittance. Notably, the *N*_bit_ = 19‐ML coating and the corresponding HMM (*N*_pair_ = 3) produced comparable interior temperatures, despite the higher visible transparency of the ML sample. This suggests that the enhanced near‐infrared reflectance (ANR) of the ML design effectively offsets the increased thermal load resulting from higher visible transmission, thereby maintaining thermal performance comparable to the darker HMM counterpart. A similar trend was observed for the thinner samples (*N*_bit_ = 13 and *N*_pair_ = 2), where the enhanced ANR of the ML coating offset the difference in visible transmittance. Together, these findings validate the experimental outperformance of ML‐driven multilayers, both optically and thermally, under practical fabrication and environmental conditions.

### 2.5 Multi‐Objective Outperformance of ML‐Driven Design

The outperformance of the ML‐driven design extends beyond the quantified optical metrics of AVT and ANR. Because the framework optimizes an arbitrary user‐defined FoM, additional objectives such as color perception can be readily incorporated, an ability that transcends the limitations of analytical multilayer design.

**FIGURE 5:**
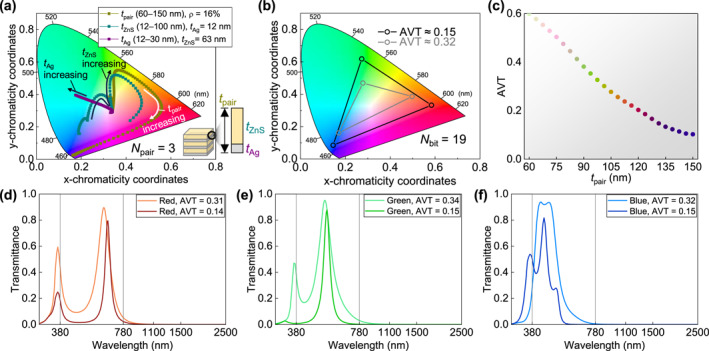
Multi‐objective ML‐driven design beyond HMM. (a) Chromaticity tuning in conventional HMMs (*N*_pair_ = 3) by varying cavity thickness tuning (*t*_pair_ = 60–150 nm, *t*_ZnS_ = 12–100 nm, *t*_Ag_ = 10–30 nm). The transmitted colors trace a narrow trajectory in the CIE color space. (b) ML‐driven designs (*N*_bit_ = 19) obtained by augmenting the FoM with a color term, reaching target chromaticities R (0.68, 0.32), G (0.21, 0.72), and B (0.14, 0.08) at AVT ∼ 0.15 (black) and 0.32 (gray). (c) Design flexibility illustrated by AVT versus *t*_pair_: HMMs show monotonically decreasing AVT with increasing *t*_pair_. (d–f) Calculated transmittance spectra of ML‐driven designs shown in (b): (d) red, (e) green, and (f) blue targets, each optimized for AVT ∼ 0.15 and 0.35. For the plots in (c) and (d–f), the marker or line colors correspond to the actual transmitted colors of each design, visually representing their realized chromaticity.

In conventional HMMs, color tuning is typically achieved by varying the dielectric cavity thickness while keeping the metal layer fixed. However, this approach intrinsically couples visible transparency and color. As shown in Figure 5a, when *t*_ZnS_ was varied from its baseline value while maintaining *t*_Ag_ = 12 nm, the transmitted colors traced a narrow trajectory in the CIE color space. Even when *t*_Ag_ and *t*_ZnS_ were varied simultaneously (i.e., sweeping *t*_pair_), maintaining high AVT with limited Ag thicknesses prevent access to blue–cyan hues (Supporting Information S1: Figure S5). Such parametric sweeps also induce undesirable effects, including shifts in the ENZ wavelength (Supporting Information S1: Figure S6).

In contrast, the ML‐driven design directly embeds color optimization into the FoM by introducing a penalty term for deviation from a target chromaticity coordinate (Figure 5b). This inverse‐design approach decouples color and transparency control, enabling simultaneous optimization of AVT, ANR, and perceived color. The resulting designs, targeted at the primary RGB coordinates (R: 0.68, 0.32; G: 0.21, 0.72; B: 0.14, 0.08), almost covered the full RGB gamut for *N*_bit_ = 19, without substantial losses in AVT or ANR.

Quantitative comparisons further highlight the distinction between HMM‐ and ML‐approaches. As shown in Figure 5c, HMMs exhibit intrinsic interdependence between AVT and chromaticity; tuning toward shorter (blue) wavelengths unavoidably reduces AVT. In contrast, the ML designs provide independent control over both parameters (Figure 5d–f), maintaining high AVT while achieving distinct chromaticity. This multi‐objective capability establishes the ML‐driven approach as a universal design paradigm, capable of realizing functionally and aesthetically optimized coatings beyond the scope of analytical multilayers (additional examples shown in Supporting Information S1: Figure S7).

## 3 Conclusion

In this work, we demonstrated the superior performance of ML‐driven inverse design for ultrathin, metal/dielectric multilayer cooling‐window coatings. By integrating a FM with SA, we identified optimized aperiodic ZnS/Ag multilayers under the same material and thickness constraints as analytical HMMs. The ML‐driven designs consistently achieved higher AVT and ANR while also enabling tunable transmitted colors covering the full visible gamut beyond the reach of HMMs. Experimental validation confirmed their superior spectral and thermal performance relative to corresponding HMM benchmarks, both through laboratory spectrophotometry and outdoor cooling tests.

Despite these advances, several limitations remain. The spontaneous formation of Ag_2_S at the interfaces causes immediate degradation in visible transmittance. This suggests the need for more stable metallic layers, such as Ag–Pd–Cu alloys [26, 27], and/or alternative high‐refractive‐index oxides (e.g., TiO_2_) to replace ZnS. We also verified that TiO_2_/Ag‐based cooling windows maintained the superior performance of the ML approach (Supporting Information S1: Figure S3). Achieving thinner (< 12 nm), continuous Ag layers that maintain percolation and consistent material dispersion also remains a challenge [28]. Scalability and practical implementation require further development; roll‐to‐roll sputtering could enable large‐area fabrication, and full‐scale evaluations on buildings or vehicles across diverse climates and latitudes are necessary to quantify real‐world energy savings. From a design perspective, emerging high‐dimensional optimization solvers such as such as quantum annealers could extend the present approach to larger material libraries, extended thickness, and finer bit discretization (more bits), thereby expanding the accessible design space [18, 19].

Overall, this study establishes ML‐driven inverse design as a powerful and versatile approach for multifunctional optical coatings, offering a pathway toward ultrathin, aesthetically tunable, and energy‐efficient cooling windows. By addressing stability, scalability, and multi‐objective optimization challenges, this framework can be extended to other nanophotonic devices requiring simultaneous control of spectral, thermal, and visual properties.

## Author Contributions

**Seok‐Beom Seo:** investigation, formal analysis, methodology, writing review and editing. **Ye‐Rin Choi:** software, formal analysis. **Jong‐Goog Lee:** software, writing review and editing. **Gumin Kang:** methodology. **Hyungduk Ko:** resources. **Run Hu:** formal analysis. **Sun‐Kyung Kim:** conceptualization, supervision, writing review and editing.

## Funding

This work was supported by the KIST institutional research program (Grant 2E33221) and the National Research Foundation of Korea through the Basic Science Research Program (Grant RS‐2023‐00207966), the Nano Material Technology Development Program (Grant 2022M3H4A1A02046445), the BK21 FOUR funded by the Ministry of Education and National Research Foundation of Korea (Grants GS‐5‐JX‐NON‐20250791 and 2120242015366), and the National Natural Science Foundation of China (52422603).

## Ethics Statement

The authors have nothing to report.

## Consent

Informed consent was obtained from all individuals included in this study.

## Conflicts of Interest

The authors declare no conflicts of interest.

## Supporting information


Supporting Information S1

## Data Availability

The data that support the findings of this study are available from the corresponding author upon reasonable request.
