# Supplementary material for: Machine Learning‐Driven Cooling Window Design Beyond Hyperbolic Metamaterials
Source: Nanophotonics. 2026 Feb 21;15(4):e70028. doi: 10.1002/nap2.70028 (PMC12964985; doi:10.1002/nap2.70028)
Supplement: Supplementary file 1 — Supporting Information S1 [file NAP2-15-e70028-s001.docx]

***Supporting Information for***

**Machine Learning-Driven Cooling Window Design Beyond Hyperbolic Metamaterials**

Seok-Beom Seo^1+^, Ye-Rin Choi^1+^, Jong-Goog Lee^1+^, Gumin Kang^2^, Hyungduk Ko^2^, Run Hu^1,3^, and Sun-Kyung Kim^1,*^

^1^Department of Applied Physics, Kyung Hee University, Yongin 17104, Republic of Korea

^2^Nanophotonics Research Center, Korea Institute of Science and Technology, Seoul 02792, Republic of Korea
^3^School of Energy and Power Engineering, Huazhong University of Science and Technology, Wuhan 430074, China
^+^These authors contributed equally to this study.
^*^Corresponding authors: sunkim@khu.ac.kr

**This PDF file includes the following sections:**

Methods

Supplement Figures S1-S8

**Methods**

**Design optimization:** In the optimization design process, we constructed a surrogate using a second-order Factorization Machine (FM) implemented in xLearn. All designs were encoded as 13, 19, 25, and 31-bit binary vectors. FM hyperparameters were fixed to latent dimension k = 12, stochastic gradient descent optimizer, learning rate 0.0001, L2 regularization 0.0001, 20,000 epochs, and no feature normalization. Early stopping was not used; after each training we computed RMSE on the training and validation sets (80/20 split at each cycle). A representative convergence and an accuracy analysis for the most challenging case (*N*_bit_ = 31) were provided in Figure S7. The reported run started from 25 initial samples and executed 2,000 optimization cycles, yielding 2,025 evaluated designs. The FM contains 404 trainable parameters (1 bias, 31 linear weights, and 31 × 12 interaction weights), which is smaller than the dataset size. Around 4 hours was taken in whole machine learning process for the most challenging case (*N*_bit_ = 31). Figure S8 demonstrates that, under this hyperparameter configuration, the FM–SA framework trains reliably and achieves stable convergence.

**Fabrication:** Machine Learning (ML)-driven and Hyperbolic metamaterial (HMM) coatings were fabricated on a glass substrate (0350-0001, LK Lab Korea), while thin films were deposited on a silicon substrate (thickness 500 ± 30 μm, Hi-Solar Co. Ltd.) for optical and material characterizations. Both glass and silicon substrates were sequentially cleaned with acetone, isopropyl alcohol, and distilled water in an ultrasonic bath. After cleaning, a soft bake at 150 °C for 10 minutes was applied to remove residual moisture and enhance adhesion. Thin films of ZnS (iTasco, 99.99%) and Ag (iTasco, 99.99%) were deposited via thermal evaporation with a deposition rate of 2 Å/s for both materials. Substrate temperature was maintained at room temperature throughout deposition. Specially, HMM samples are initially treated with 1nm Ti deposition for enhancing Ag/SiO_2_ adhesion.

**Optical characterization:** The refractive indices of ZnS and Ag thin films were measured across visible and near-infrared wavelengths using ellipsometry (Elli-SE-DU, Ellipso technology Co.). For refractive index measurements, Ag and ZnS thin films were individually deposited on glass substrates as single layers with nominal thickness of 12 nm and 63 nm, respectively. The transmittance spectrum of the cooling windows was obtained using a spectrophotometer (Cary 5000, Varian). Cross-sectional TEM images of the samples were acquired using an HR-TEM (JEM-2100F, JEOL Co.). Samples are prepared with FIB dicing (NOVA Nanolab, FEI Co.) with 1 micrometer size.

**Outdoor measurements:** Outdoor measurements were conducted on the open field near the College of Electronics & Information building at Kyung Hee University, Yongin-si, Gyeonggi-do, South Korea (coordinates: 37.23983° N, 127.08371° E). Thermocouples (SA1-K-SC, Omega™) logged real-time sample temperatures via a (specify model) temperature logger. An indoor simulator was constructed with a PLA body, black insulating tape, and aluminum tape for sunlight reflection; PLA parts were 3D-printed.

**Figure S1. Refractive indices of candidate materials directly measured by spectroscopic ellipsometry.** (a) Refractive indices of ZnS and Ag thin films. (b) Effective refractive index calculated from effective medium theory with an Ag filling fraction of ρ = 16%.


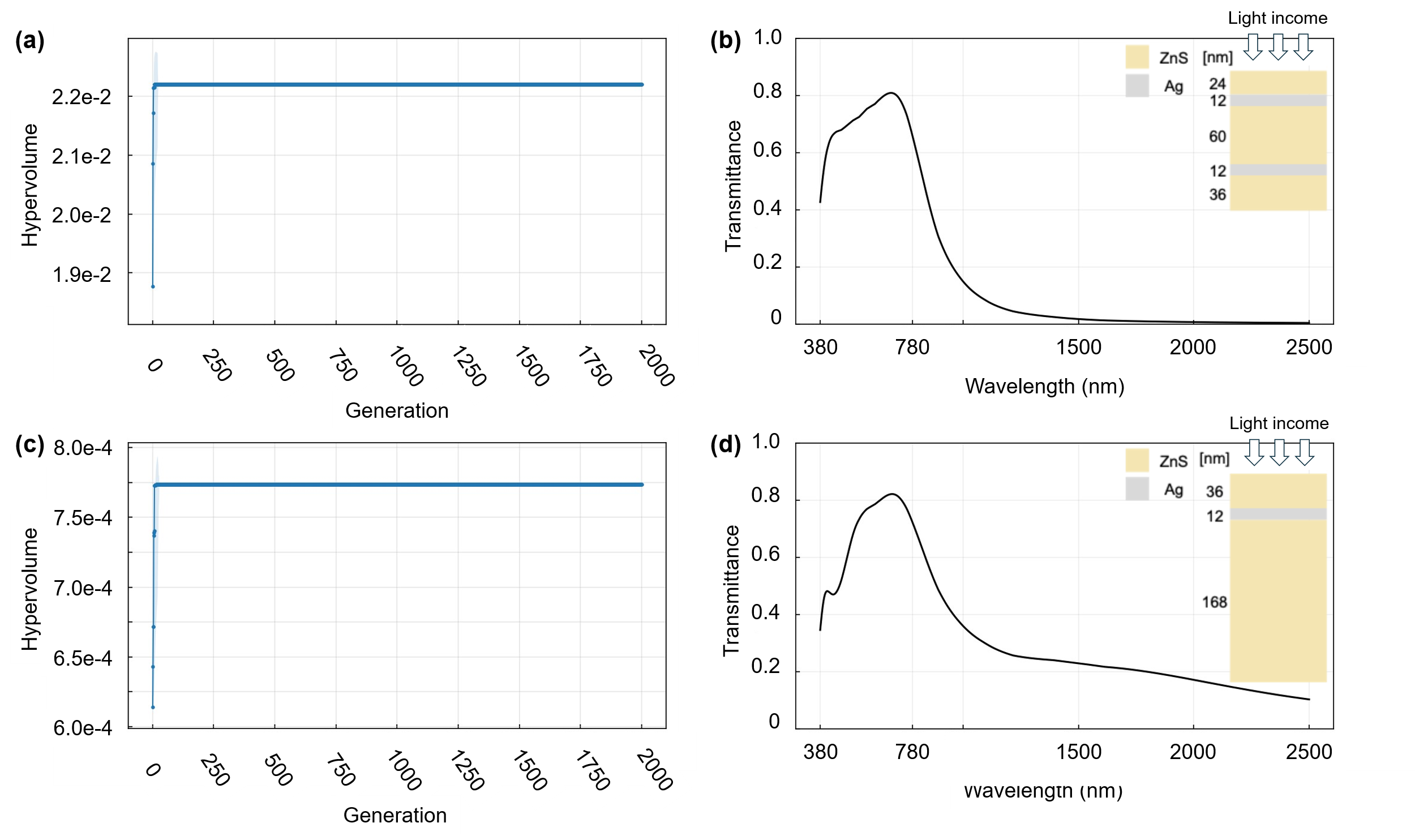


Wavelength (nm)

**Figure S2. Optimization results using the Non-dominated Sorting Genetic Algorithm II (NSGA-II) algorithm for ZnS/Ag multilayers.** To investigate whether a simpler heuristic optimizer can approach the performance of the proposed ML framework, we applied the NSGA-II to the same design problem. **(a, c)** Evolution of the hypervolume indicator as a function of generations for the **(a)** *N*_bit_ = 13 and **(c)** *N*_bit_ = 19 design cases. The saturation (plateau) of the hypervolume curves indicates that the population has successfully converged to the approximate Pareto front. The shaded narrow, light-colored band represents the standard deviation of the hypervolume over ±20 generations around each data point, with larger values indicating more active search dynamics. **(b, d)** Calculated transmittance spectra and corresponding layer configurations of the best-performing designs obtained from NSGA-II for **(b)** *N*_bit_ = 13 and **(d)** *N*_bit_ = 19. The resulting designs exhibit spectral performance and structural features comparable to those obtained via the FM-SA approach, demonstrating that standard genetic algorithms can also reach similar solution qualities in this material system. Inset of (b,d): structural information of (b) *N*_bit_ = 13 and (d) *N*_bit_ = 19 design.

**Figure S3.** **Performance metrics of designed (patterned bar) and fabricated (solid bar) benchmark candidates.**

**Figure S4. Alternative Hyperbolic metamaterials (MMM) and machine learning (ML)-driven cooling window designs employing TiO_2_ cavities.** (a) Transmittance spectra of HMM-based cooling windows with *N*_pair_ = 2, 3, 4, and 5. (b) Transmittance spectra of ML-driven cooling windows with *N*_bit_ = 13, 19, 25, and 31. Both design groups shave identical thickness parameters (*t*_Ag_ = 12 nm, *t*_bit_ = 12 nm, *t*_TiO2_ = *t*_ZnS_ = 63 nm, *t*_pair_ = 75 nm.). (c) Quantitative comparison of AVT, ANR, and FoM between ML and HMM designs under identical thickness constraints.

**Figure S5.** **Variation of AVT and ANR as functions of *t*_ZnS_ (dark cyan), *t*_Ag_ (violet), and *t*_pair_ (olive) with the *N*_pair_ = 3 HMM.** Parametric sweep calculations are conducted over ranges of 12–100 nm for *t*_ZnS_, 10–30 nm for *t*_Ag_, and 60–150 nm for *t*_pair_.

**Figure S6. Transmittance spectra of the *N*_pair_ = 3 HMM under parametric sweeps (ranges identical to Fig. S5).** (a) *t*_ZnS_ sweep: increasing the dielectric-cavity thickness increases the overall Fabry–Perot cavity length, shifting resonance peaks/deeps toward longer wavelengths. In addition, because the effective (averaged) permittivity changes within the effective-medium picture, the ENZ point and the transmission-blocking edge shift accordingly. (b) *t*_Ag_ sweep: increasing the metal thickness primarily reduces the overall transmittance level (stronger attenuation/reflectance), and simultaneously changes the effective permittivity, leading to an ENZ shift and thus a change in the cutoff (blocking) wavelength. (c) *t*_pair_ sweep at a fixed thickness ratio (constant filling fraction): scaling both metal and dielectric thicknesses together largely preserves the ENZ wavelength, while the two effects above occur simultaneously within this ENZ-fixed condition—i.e., the resonance positions shift due to the increased optical cavity thickness, and the overall transmittance decreases due to the thicker metal. Overall, these trends indicate that parametric tuning in periodic HMMs does not provide an intuitive, independent handle for selecting a desired transmitted color and typically inevitably accompanies a change in visible transmittance (AVT).

**Figure S7. Additional color-tuning results for different target AVT values across various *N*_bit_ ML-driven designs.** During the design process, *N*_bit_ = 19 designs targeted AVT values of 0.15 (bottom) and 0.35 (top); *N*_bit_ = 25 designs targeted AVT values of 0.15 (bottom), 0.25 (middle), and 0.35 (top); *N*_bit_ = 31 designs targeted AVT values of 0.15 (bottom), 0.25 (middle), and 0.5 (top). The symbols are filled with their corresponding perceived transmitted colors. Because the color target enters the FoM through the target chromaticity coordinate, diverse hues can be obtained at a comparable AVT by modifying only the target chromaticity in the FoM. Likewise, by setting the target AVT while keeping the color target fixed, AVT can be adjusted while maintaining the desired transmitted color objective (within the accessible solution space). The derived hues exhibit a clear trend in which higher AVT correlates with diminished color saturation, reflecting the inherent interdependence between transparency and chroma. Such flexible, multi-objective control is generally difficult to obtain via simple parametric thickness tuning of periodic HMMs, highlighting the advantage of the ML inverse-design approach. The upper boundary of the AVT distribution does not represent a theoretical transmittance limit.


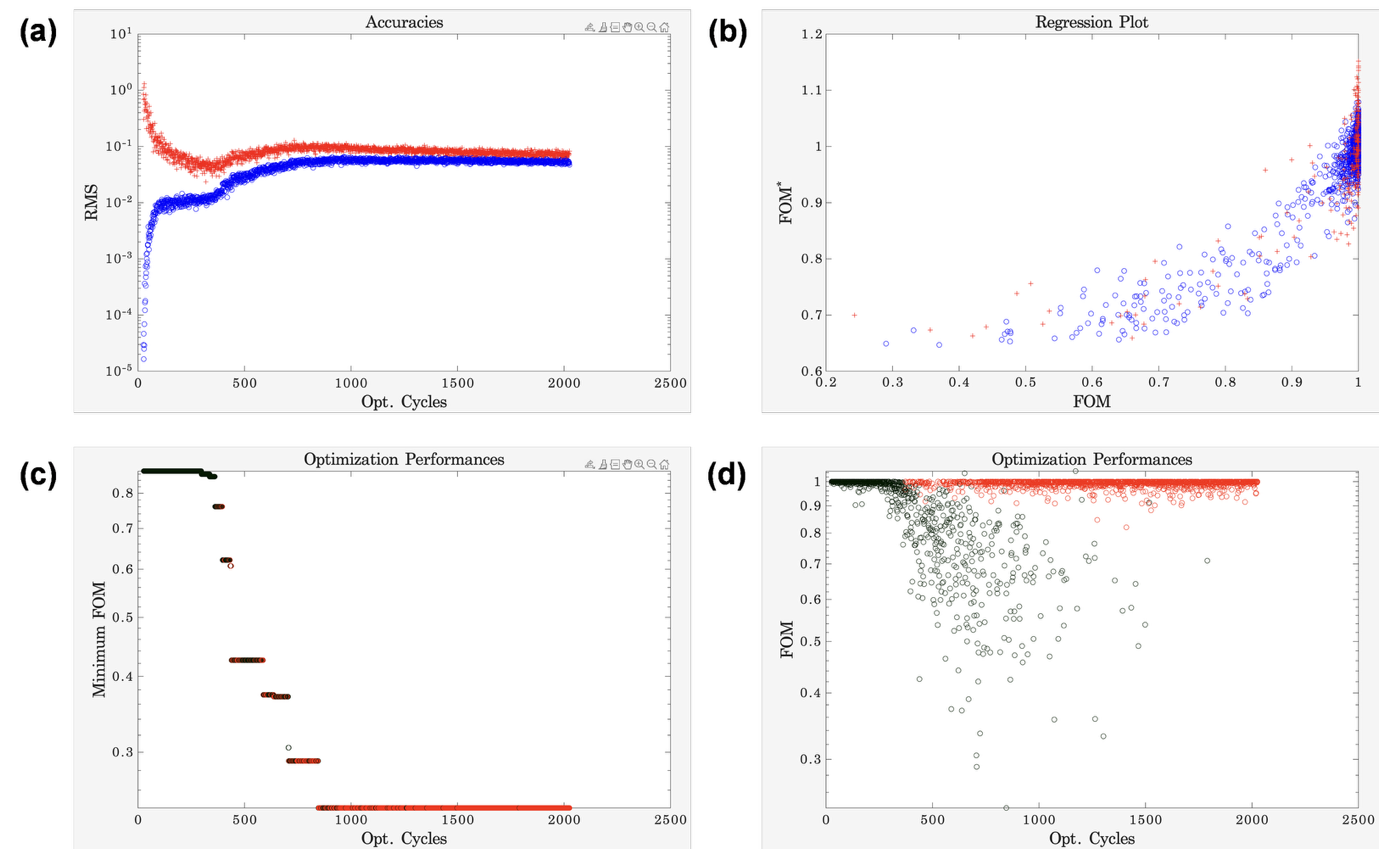


**Figure S8. Active-learning convergence and surrogate accuracy for the *N*_bit_ = 31 case.** (a) RMSE evolution versus optimization cycles (computed on the training/validation split), showing stabilization as the dataset grows. (b) Regression between exact FoM (x-axis; physics solver) and FM-predicted FoM* (y-axis) for the training/validation split at a representative cycle. (c) Best-so-far (minimum) FoM as a function of optimization cycles, showing stepwise improvement and convergence after ~800–900 cycles. (d) FoM of proposed/evaluated designs versus optimization cycles; red markers indicate duplicate proposals (the inferred digital vector was identical to a previously evaluated one).
